# Effect of Marination on the Formation of Polycyclic Aromatic Hydrocarbons in Grilled Vegetables

**DOI:** 10.1002/fsn3.70600

**Published:** 2025-07-07

**Authors:** Sibel Kacmaz Ozcetin, Levent Artok

**Affiliations:** ^1^ Department of Food Engineering, Engineering Faculty Giresun University Giresun Türkiye; ^2^ Department of Chemistry, Faculty of Science Izmir Institute of Technology Izmir Türkiye

**Keywords:** grilled vegetables, inhibition, marinated vegetables, marination, natural antioxidants, PAH

## Abstract

The effect of marination on the formation of polycyclic aromatic hydrocarbons (PAH) in charcoal‐grilled vegetables was studied. Various marinade ingredients, including apple cider vinegar, red grape vinegar, lemon juice, garlic powder, black pepper, and the food additive *tert*‐butylhydroquinone (TBHQ) were applied to vegetable samples before charcoal grilling. The total phenolic content (TPC) and total antioxidant capacity (TAC) of each marinade ingredient were assessed for their contribution to PAH inhibition. A substantial decrease in PAH4 formation was observed in marinated vegetables. Red grape vinegar exhibited the strongest average inhibitory effect on total PAH4 formation (75%), followed by apple vinegar (68%), lemon juice (52%), garlic powder (34%), and black pepper (30%). Additionally, the TBHQ (67%) demonstrated a strong inhibitory effect, reducing total PAH4 formation by 67%. These findings offer valuable insights for reducing PAH levels in grilled vegetables and preventing their formation.

## Graphical abstract

The effect of marination on the formation of polycyclic aromatic hydrocarbons (PAH) in charcoal‐grilled vegetables was studied. A substantial decrease in PAH4 formation was observed in marinated vegetables. These findings offer valuable insights for reducing PAH levels in grilled vegetables and preventing their formation.

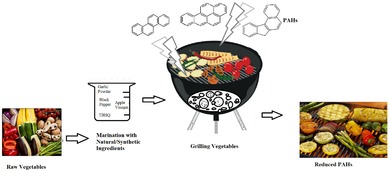

## 1 Introduction

Polycyclic aromatic hydrocarbons (PAHs) are potentially harmful substances of global concern due to their carcinogenic, genotoxic, and mutagenic properties. They are produced through pyrolysis and incomplete combustion of organic matter, primarily released during food cooking. Several global organizations, including the Joint FAO/WHO Expert Committee on Food Additives (JECFA), the Scientific Committee on Food (SCF), and the International Agency for Research on Cancer (IARC), have recognized PAHs as key hazardous compounds (SCF 2002a, 2002b; European Commission (EC) 2005; CCFAC 2005; JECFA 2005; IARC 2010, 2012). While many PAHs exist, PAH4 (BaP; Benzo[a]pyrene, BbF; Benzo(b)fluoranthene, CHR; chrysene, and BaA; Benz[a]anthracene) has been recognized by the EFSA (European Food Safety Authority) as the most relevant indicators based on evaluation of toxicity (EFSA 2008a, 2008b).

Vegetables are a vital component of the human diet worldwide, thanks to their rich nutritional content. Raw vegetables generally contain low levels of PAHs (Camargo and Toledo 2003; Ashraf and Salam 2012; Ashraf et al. 2013; Paris et al. 2018; Kacmaz et al. 2023), which accumulate on their surfaces primarily due to PAH‐rich particles present in the air and soil (SCF 2002a, 2002b). Although some vegetables are consumed raw, most are prepared using various cooking methods such as grilling, baking, frying, and roasting. It is evident that cooked vegetables also contribute to human PAH exposure (Alomirah et al. 2011; Akpambang et al. 2015; Cheng et al. 2019). Grilling, an ancient and popular cooking method using strong direct radiant heat or charcoal, is still widely used for both meats and vegetables. However, direct contact between food and the heat source significantly increases the risk of PAH formation and concentration (Kazerouni et al. 2001; Cheng et al. 2019).

Marinating is a widely used technique to enhance the sensory properties of foods, such as texture, color, and flavor prior to grilling. However, its effect on PAH formation can vary depending on the ingredients used. Marination has emerged as a new strategy to control and reduce PAH formation in charcoal‐grilled foods, including beef (Farhadian et al. 2012; Kılıç Büyükkurt et al. 2020), chicken wings (Wang et al. 2018, 2019a, 2019b), duck meat (Sinaga et al. 2016), pork (Viegas et al. 2014; Cordeiro et al. 2020), and other meat products (Das et al. 2023). Studies have shown that marinating with ingredients like beer, red or white wine, tea, lemon juice (LJ), vinegar, black garlic, and seasonings can effectively reduce PAH levels.

However, there is limited information on PAHs through marinating in grilled vegetables. This study sought to investigate the inhibitory effects of marinating ingredients on PAH formation in charcoal‐grilled vegetables. Vegetables were marinated with natural antioxidant‐rich components and antioxidant food additives, such as *tert*‐butylhydroquinone (TBHQ), before charcoal grilling. The effectiveness of each marinade in inhibiting PAH formation was evaluated. Additionally, the total phenol content (TPC), total antioxidant capacity (TAC), and PAH inhibition efficiency of each marinade were assessed. GC/MS was utilized to assess PAHs in vegetables before and after marinating. This is the first report about the PAH inhibition efficiency of marinating ingredients on charcoal‐grilled vegetables.

## 2 Materials and Methods

### 2.1 Reagents and Standards

The mixed PAH calibration solution, which included BaA, CHR, BbF, and BaP (10 μg/mL in toluene), was supplied by the Institute for Reference Materials and Measurements (IRMM) in Geel, Belgium. It was prepared using toluene in concentrations varying from 0.5 to 85 μg/kg. All solvents utilized for extraction and analysis, including ethanol, acetone, acetonitrile (99.9%), and cyclohexane (> 99.5%), were of HPLC grade and supplied from Sigma (Steinheim, Germany). Folin–Ciocalteu reagent, gallic acid, 1,1‐diphenyl‐2‐picrylhydrazyl (DPPH), anhydrous sodium sulfate, and sodium carbonate were obtained from Merck (Darmstadt, Germany). The used SPE cartridges (50 μm particle size, 500 mg/4 mL) were from GRACE (Grace Davison Discovery Science, Columbia, MD, USA), and PTFE acrodisk syringe filters (Polytetrafluoroethylene, 25 mm i.d., 0.45 μm) were supplied from Sigma Aldrich.

### 2.2 Sample Preparation, Marinating and Charcoal Grilling

Vegetables commonly used for charcoal grilling, including potato, eggplant, bottle gourd, onion, red pepper, and green pepper, were purchased in October 2023 from a vegetable market in İzmir, Türkiye. Each sample was immediately transported to the laboratory and stored at +4∘C until analysis. The raw vegetable samples were first washed with tap water, followed by deionized water, and then chopped into approximately 1.0 cm before marination. Untreated (raw) vegetables were found to have low concentrations of PAHs (Kacmaz et al. 2023).

Marinating ingredients such as apple vinegar (AV), red grape vinegar (RV), garlic powder (GP), black pepper powder (BP), LJ, along with food additives like TBHQ were tested. The total phenolic content (TPC) and DPPH free radical scavenging activity were measured for each ingredient. Vegetable samples were marinated with each of the marinades and stored in a refrigerator at 4°C. Marinating treatments of varying durations were not tested, as marinating time was not found to be a significant factor (*p* > 0.05) in PAH inhibition in studies conducted by other researchers (Farhadian et al. 2012; Inbaraj et al. 2024; Singh et al. 2023). The amount of marinating ingredients for 100 g of vegetables is shown in Table 1. Thirty‐six samples were used for each vegetable: four independent samples for each marinade compound, and four control samples (Untreated).

**TABLE 1:** Ingredients and their quantities for marinating 100 g of vegetables.

Ingredients	Amount of marinating ingredients
Red grape vinegar (RV)	2 mL
Apple vinegar (AV)	2 mL
Black papper (BP)	0.6 g
Garlic powder (GP)	1.5 g
Lemon juice (LJ)	2 mL
*tert*‐Butylhydroquinone (TBHQ)	20 mg (0.02%)

The marinated vegetable samples were charcoal‐grilled using a garden‐type barbecue grill (80 cm wide, 60 cm long, and 60 cm high). After extinguishing the open flame, the vegetable and the control samples were cooked over hot charcoals (placed away 20 cm from the charcoals) for 5–8 min, depending on the vegetable type. Specifically, potatoes were grilled for 8 min, eggplants for 7 min, bottle gourds for 6 min, onions for 7 min, and both red and green peppers for 5 min. The samples were rotated every 2 min during the grilling process. A digital thermocouple featuring a surface probe (Testo 926 TE type T, Germany) was utilized to measure the core temperatures of the charcoal and vegetables during the grilling process, reaching temperatures of approximately 220°C and 50°C, respectively. After grilling, the vegetables were homogenized and stored at −80°C until PAH analysis.

### 2.3 Determination of Total Phenolic Content

The TPC of each marinating ingredient was assessed using the Folin–Ciocalteu method, following the protocols outlined by Singleton et al. (1999) and Altiok et al. (2022), with gallic acid as the standard. Briefly, 0.5 mL of the diluted sample (1:10) was combined with 2.5 mL of diluted Folin–Ciocalteu reagent (1 N) and allowed to stand for 2.5 min at rt. Subsequently, 1.25 mL of saturated Na_2_CO_3_ solution (7% in deionized water) was added. The absorbance was measured at 725 nm using a UV–Visible spectrophotometer (Mapada Uv‐6100 psc) after a 60‐min incubation in the dark. The measured values were calculated based on a calibration curve constructed with gallic acid (GA) at concentrations ranging from 0 to 80 mg/L. The TPC was expressed as milligrams of gallic acid equivalents (GAE) per liter.

### 2.4 Determination of the Total Antioxidant Capacity

The TAC of each marinating ingredient was evaluated using the DPPH assay method as described by Brand‐Williams et al. (1995), Singh et al. (2002), and Wang et al. (2018). A 200 μL aliquot of each marinating ingredient, diluted at a ratio of 1:10, was mixed with an equal volume of DPPH (0.2 mM, dissolved in 95% ethanol). The control sample (*A*_control_) was prepared by mixing equal volumes (200 μL each) of DPPH and 95% ethanol without the addition of any marinating ingredient. The blank (*A*_blank_) was prepared using equal volumes (200 μL each) of DPPH and distilled water. After the samples were kept in the dark for 30 min, the absorbance at 517 nm was recorded using UV–vis absorbance spectroscopy. Measurements were performed in triplicate. The radical scavenging effects of the antioxidant components present in the sample were calculated using the formula:

$$\text{DPPH scavenging activity}\;\left(\%\right)=\left[1-\frac{\left(A_{\text{sample}}\left.-A_{\text{blank}}\right)\right.}{\left(A_{\text{control}}\left.-A_{\text{blank}}\right)\right.}\right]\times 100$$

### 2.5 PAH Analysis

#### 2.5.1 Extraction of PAHs

The extraction and cleanup procedures for vegetables were carried out following the method outlined by Ashraf et al. (2013), Ashraf and Salam (2012), Paris et al. (2018) and Kacmaz et al. (2023) with slight modifications. Briefly, each vegetable sample (50 g) was homogenized and extracted using a Soxhlet extractor with a solvent mixture of acetone: cyclohexane (1:2 v/v) for 4 h. The extracts were filtered through 125 mm filter paper, which was packed with 100–150 g of preheated anhydrous sodium sulfate, based on the water content of the extract.

The filtrate was then evaporated to dryness under a nitrogen at a temperature of 40°C. Afterward, the dry extract was reconstituted in 1 mL of cyclohexane and filtered using 1 μm PTFE syringe filters. The silica SPE cartridge was used for sample purification. Initially, the SPE cartridge was conditioned with 2 mL of cyclohexane, and the extract was purified by passing 10 mL of cyclohexane through the SPE column. The collected SPE fraction was then combined with 200 μL of toluene as a “keeper” and then concentrated to approximately 200 μL at 40°C. The resulting residue was rinsed with an additional 200 μL of toluene and transferred to the GC vial.

#### 2.5.2 GC–MS System

PAH quantification was carried out using a GC–MS system, comprising a Gerstel MPS‐Agilent 5977C GC/MSD 8890 GC System and an Agilent 6890N/5973N Thermo Scientific Trace GC Ultra PAH separations were performed using an analytical column with a length of 15 m, an internal diameter of 0.15 mm, and a film thickness of 0.10 μm (provided by Agilent Technologies) (Kacmaz 2016; Kacmaz et al. 2016).

#### 2.5.3 Method Validation

The analytical method was in‐house validated for several parameters, including the limit of detection (LOD), limit of quantification (LOQ), selectivity, precision, linearity, recovery values with relative standard deviation (RSD), and HORRAT values, as well as measurement uncertainty. Validation was conducted in accordance with the Commission Directive (European Commission (EC) 2011) and Eurachem guidelines (Eurachem/Citac Guide 2000; Eurachem 2014). Nine calibration curves were generated using a standard mixture of the four PAHs, with concentrations ranging from 0.4 to 85 μg/kg. The linearity was verified using Mandel's test (Mandel 1964). LOD and LOQ were calculated using the formulas LOD = 3.3*σ*/*s* and LOQ = 10*σ*/*s* where *σ* represents the standard deviation of responses from replicate analyses (*n* = 10); and *s* is the slope of the calibration curve. Precision was evaluated through ANOVA, expressed as repeatability (RSD_r_%) and intermediate precision (RSD_IP_%). The measurement uncertainty was estimated using the error propagation law in accordance with the Eurachem/Citac guidelines. This approach combines uncertainties from various sources, including PAH standard solutions, the calibration curve, precision, and bias (Eurachem 2014; Kacmaz et al. 2016; Sibel Kacmaz 2019).

## 3 Results and Discussion

### 3.1 Single Laboratory Validation

Validation studies indicated that all PAH compounds demonstrated excellent linearity, with *R* ^2^ values exceeding 0.99. LOD and LOQ for the vegetable matrix were determined to be within the range of 0.02–0.03 μg/kg and 0.03–0.05 μg/kg, respectively (see Table 2). Precision was assessed using vegetables spiked with 0.25 μg/kg of PAH standard solution and was expressed as the relative standard deviation of repeatability (RSDr%) and the relative standard deviation of intermediate precision (RSD_IP_%). The RSDr and RSD_IP_ values ranged from 6.7% to 11%, which are lower than the thresholds of 15% and 20%, respectively, for all analytes that meet the criteria (Table 2). Recovery experiments were conducted using five replicate samples of 50 g of mixed vegetables, each spiked with 1 μg/kg PAHs. The average recovery rates, along with their average standard deviation (SDs), ranged from 78% ± 8% to 90% ± 5%. Additionally, the HORRAT values were found to range between 0.05 and 0.07, in accordance with the EC regulation (EC 2011), which defines the precision performance criteria for methods as HORRAT < 2 for precise analysis. The expanded uncertainty (U) of the validated technique was calculated using data for 0.1 μg/kg of PAHs as the combined uncertainty, with a coverage factor (*k*) of 2 at a 95% confidence level. The expanded uncertainty was expressed as being less than 20% (Table 2).

**TABLE 2:** Limits of detection (LODs), limits of quantification (LOQs), HORRAT values for repeatability (HORRAT_r_) and reproducibility (HORRAT_R_), relative standard deviation of repeatability (RSD_r_), and intermediate precision (RSD_IP_), mean recoveries (with standard deviations, SD%), and measurement uncertainty (U) for PAH4 in vegetable samples (spiked with 1 μg/kg of each of the four analyte).

PAHs	BaA	CHR	BbF	BaP
LOD (μg/kg)	0.02	0.02	0.02	0.03
LOQ (μg/kg)	0.03	0.03	0.03	0.05
RSD_r_ (%)	6.7	6.9	11.0	8.0
RSD_IP_ (%)	6.7	6.9	11.0	10.7
HOR_r_	0.6	0.6	0.7	0.6
HOR_R_	0.5	0.5	0.5	0.7
Recovery ± SD (%)	89 ± 6	86 ± 5	78 ± 8	90 ± 5
U (*k* = 2) (%)	13	20	13	17

### 3.2 Total Phenolic Content and Antioxidant Activities of Marinating Ingredients

**FIGURE 1:**
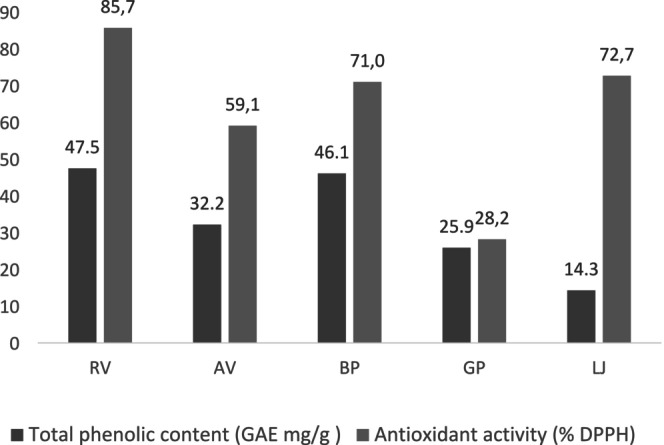
Total phenolic content and antioxidant activity of each marinade ingredients. AV, apple vinegar; BP, black papper; GP, garlic powder; LJ, lemon juice; RV, red grape vinegar.

The TPC and antioxidant activities of each marinating ingredient were investigated. As shown in Figure 1, all marinades demonstrated a high TPC and displayed significant DPPH radical scavenging activity. The TPC values ranged from 47.5 ± 3.4 to 14.3 ± 0.4 mg GAE/g. RV exhibited the highest TPC at 47.5 ± 3.4 mg GAE/g, followed by black pepper (46.1 GAE/g), AV (32.2 GAE/g), GP (25.9 GAE/g), and LJ (14.3 GAE/g). Phenolic compounds are essential natural components that significantly affect the aroma, taste, and color of foods, while also playing a crucial role in their antioxidant activity. The antioxidant properties of each marinating ingredient were also evaluated, with RV showing the highest antioxidant activity (85.7%), followed by LJ (72.7%), black pepper (71.0%), AV (59.1%), and GP (28.2%).

### 3.3 Effect of Marinating Ingredient on PAH Formation in Vegetables

Six different vegetables—potato, eggplant, bottle gourd, onion, red pepper, and green pepper—were used to assess the effect of marinating on the formation of PAHs. To accomplish this, PAH content was measured in grilled vegetables treated with various ingredients, as well as in control samples that underwent no treatments.

Table 3 presents the concentrations of PAH4 in the control samples, which consist of grilled vegetables without any added ingredients. The four PAHs analyzed were found to range from 7.52 ± 3.36 to 21.79 ± 3.33 μg/kg of wet weight. Among the grilled vegetables, green peppers stand out with the highest total PAH4 content (21.79 ± 3.33 μg/kg), followed by potatoes (16.39 ± 1.05 μg/kg), bottle gourd (13.21 ± 1.85 μg/kg), eggplant (10.95 ± 1.85 μg/kg), red pepper (8.15 ± 1.23 μg/kg), and onion (7.52 ± 3.36 μg/kg). In the analysis, Benz[a]anthracene (BaA) was found to be the highest among the four genotoxic PAH compounds in grilled vegetables, while benzo[a]pyrene (BaP), the most representative carcinogenic PAH compound, was detected at the lowest levels in all samples. The differences in PAH content among the grilled vegetables may be attributed to several factors, including variations in the composition of each vegetable, changes in surface area, and the amount of plant cellulose, which can contribute to PAH formation through a series of chemical transformations during pyrolysis at high temperatures (Zachara et al. 2017; Camargo and Toledo 2003; McGrath et al. 2007; Cheng et al. 2019). Furthermore, due to the unique antioxidant properties of raw vegetables (Benzie and Wachtel‐Galor 2011; Lu et al. 2011; Kim et al. 2019), it is highly plausible that they exert inhibitory effects on PAH formation during heat processing.

**TABLE 3:** Concentration (μg/kg ± SD) of PAHs in grilled vegetables.

Vegetables	BaA	CHR	BbF	BaP	ΣPAH4
Potato	8.63 ± 2.76	4.29 ± 3.31	1.53 ± 1.66	1.94 ± 1.24	16.39 ± 1.05
Eggplant	8.39 ± 2.31	2.30 ± 1.06	0.11 ± 0.10	0.15 ± 0.10	10.95 ± 1.85
Bottle gourd	3.84 ± 1.58	4.85 ± 2.36	3.31 ± 0.70	1.21 ± 0.65	13.21 ± 1.85
Green pepper	10.32 ± 2.3	5.03 ± 1.83	2.75 ± 1.38	3.69 ± 1.47	21.79 ± 3.33
Red pepper	3.20 ± 1.70	2.01 ± 1.16	1.75 ± 0.38	1.19 ± 0.89	8.15 ± 1.23
Onion	2.90 ± 1.20	2.40 ± 1.11	1.4 ± 1.2	0.82 ± 0.03	7.52 ± 3.36

*Note:* Results are presented as the mean ± SD, *n* = 4.

Abbreviations: BaA, Benz[a]anthracene; BaP, benzo[a]pyrene; BbF, benzo[b]fluoranthene; CHR, chrysene; PAH4, four European Union (4EU) priority polycyclic aromatic hydrocarbons.

The total PAH4 concentration in grilled vegetables (Potato: 16.39 ± 1.05 μg/kg, Eggplant: 10.95 ± 1.85 μg/kg, Bottle gourd:13.21 ± 1.85 μg/kg) reported in this study was approximately 25–50 times higher than that found in raw vegetables in our previous study (Potato: 0.73 μg/kg, Eggplant:0.34 μg/kg, Bottle gourd: 0.28 μg/kg) (Kacmaz et al. 2023). Similar results were reported by Alomirah et al. (2011) and Martí‐Cid et al. (2008). The significant increase in PAH levels in grilled vegetables, as observed in other charcoal‐grilled foods, is primarily due to the incomplete combustion of charcoal, the direct contact with the grill, and the pyrolysis of fat that is dripping onto the heat source.

**FIGURE 2:**
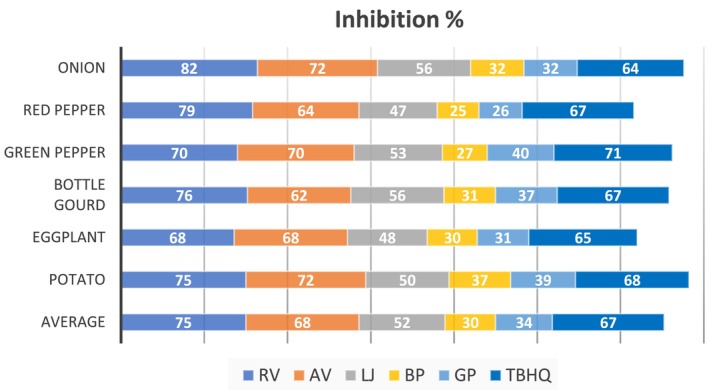
Average reduction by each marinade ingredient. AV, apple vinegar; BP, black pepper; GP, garlic powder; LJ, lemon juice; RV, red grape vinegar; TBHQ, *tert*‐butylhydroquinone.

Marinating vegetables with various ingredients before grilling significantly reduces the total PAH4 concentrations. The average reduction for each marinade ingredient is illustrated in Figure 2. Among the ingredients studied, RV and AV showed the strongest inhibitory effects on the formation of total PAH4, with reductions ranging from 68% to 82% and 62% to 72%, respectively. This effectiveness is likely due to their high levels of phenolic compounds and strong free radical scavenging capacity, which can interrupt the formation of PAHs by neutralizing reactive intermediates generated during high‐temperature cooking. Similar results were also found in studies of charcoal‐grilled pork (Cordeiro et al. 2020) and barbecued pork sausages (Zhang et al. 2023). In the study of Cordeiro et al. (2020) the highest inhibition of PAH4 formation in pork samples sprayed with vinegar prior to charcoal grilling was observed with elderberry vinegar (82%), followed by white wine vinegar (79%), red wine vinegar, and cider vinegar (66%), and raspberry juice‐based fruit vinegar (55%). Additionally, Zhang et al. (2023) reported that fermented vinegars and distilled vinegar significantly inhibited the formation of BaP (26.8%–82.3%) in barbecued pork sausages (*p* < 0.05).

LJ exhibited an inhibitory effect on the formation of total PAH4, with reductions in the range from 47% to 56%. It has been observed that marinating vegetables with LJ has a positive effect in inhibiting PAHs. The alteration in acidic content (lower pH) of samples marinated with LJ may affect the kinetics and mechanism of Maillard reactions. Specifically, the lower pH may suppress Maillard‐driven pathways that contribute to PAH formation, as these reactions tend to proceed more rapidly under higher pH conditions (Brien et al. 1998) which could have contributed to the reduction of PAH concentrations. Research on the mechanisms and kinetics of PAHs has shown that these compounds play a role in the aroma profiles produced during Maillard reactions (Britt et al. 2004). However, the complex nature of these reactions means the full extent of their relationship with PAHs remains unclear. Nonetheless, other Maillard products and Amadori compounds can break down into substances that contribute to the distinctive aroma and flavor of roasted and baked products, while also creating the typical brown color of cooked foods (Parliament et al. 1994; Farhadian et al. 2012).

Marinating with natural antioxidants, such as GP and black pepper, efficiently reduced PAH formation in vegetables by 26%–39% and 25%–37%, respectively. This is likely associated with their antioxidant components, such as organosulfur compounds and polyphenols, which may inhibit PAH formation by scavenging free radicals and blocking oxidative pathways during grilling. Similar results were reported for various natural antioxidant marinades and different types of food products by Gong et al. (2018), Lu et al. (2018), Wang et al. (2022), and Tian et al. (2023). In the study by Tian et al. (2023), curcumin significantly inhibits PAHs (16%–72%) in pre‐marinated chicken wings. Additionally, research on spice‐based marinating (onion, garlic, paprika, red chili, and ginger at 0.5% powder) reported reductions in B(a)A and B(a)P in beef and chicken meatballs by 47.02%–97.9% and 74.8%–97.2%, respectively (Lu et al. 2018).

Additionally, TBHQ, a commercial additive commonly used to preserve processed foods, was used as a marinating ingredient in this study and inhibited PAH formation in grilled vegetables by 64%–71%. A similar efficient reduction (71.75%) was reported by Zhao et al. (2017), where the inclusion of TBHQ in the frying oil notably lowered the levels of PAHs and OPAHs in fried peanuts. In a study by Gong et al. (2018), increasing the antioxidant concentration from 60 to 180 μg/kg resulted in a 20%–40% reduction in total PAH content across various groups, with TBHQ showing the most effective inhibitory impact.

Taken together, these findings not only contribute to the understanding of chemical mechanisms involved in PAH reduction such as free radical scavenging, pH modulation, and antioxidant action—but also offer practical implications for home cooks and the food industry. The results indicated that marinating vegetables with various ingredients before charcoal grilling can effectively reduce PAH formation. Moreover, marinating vegetables before grilling could enhance sensory attributes such as tenderness, flavor, juiciness, and overall acceptability. Incorporating natural marinades rich in polyphenols and acids, or using approved synthetic antioxidants, may represent a feasible and consumer‐friendly strategy to reduce PAH exposure in grilled foods, while simultaneously improving the taste and quality of the final product.

## 4 Conclusion

This study explored the effectiveness of marination in inhibiting PAH formation in charcoal‐grilled vegetables. The TPC and antioxidant activity of each marinating ingredient were assessed. Our findings reveal that marinating resulted in an average reduction in PAH4 levels, ranging from 30% to 75%. RV was the most effective, reducing PAH4 levels by 75%. Other effective marinades included AV (68%), LJ (52%), GP (34%), and black pepper (30%), all of which significantly inhibited PAH formation. Similarly, TBHQ, a food preservative, showed a comparable effect with a 67% reduction.

The use of natural marinating ingredients rich in phenolic compounds and antioxidant activity can significantly reduce the formation of PAH4 in charcoal‐grilled vegetables. Additionally, antioxidant food additives can also be effectively used as marinating ingredients.

## Author Contributions

**Sibel Kacmaz Ozcetin:** conceptualization (lead), formal analysis (lead), investigation (lead), methodology (lead), project administration (equal), validation (lead), visualization (lead), writing – original draft (lead). **Levent Artok:** funding acquisition (equal), supervision (equal), visualization (equal), writing – review and editing (equal).

## Ethics Statement

The authors have nothing to report.

## Conflicts of Interest

The authors declare no conflicts of interest.

## Data Availability

The data that support the findings of this study are available from the corresponding author upon reasonable request.
